# Prospective evaluation of the diagnostic accuracy of FDG-PET/CT for the detection of sternal wound infection post-sternotomy

**DOI:** 10.1186/s41824-024-00237-1

**Published:** 2025-02-08

**Authors:** Angela Cai, Mehrshad Bakhshi, Yoan Lamarche, Francois Harel, Matthieu Pelletier-Galarneau

**Affiliations:** 1 Department of Medical Imaging, Montreal Heart Institute, Montreal, QC H1T 1C8 Canada; 2 Department of Surgery, Montreal Heart Institute, Montreal, QC Canada

**Keywords:** Sternal wound infection, FDG-PET/CT, Deep sternal wound infection, Sternotomy, Infection imaging

## Abstract

**Purpose:**

Sternal wound infections (SWI) are complications of sternotomy and can be divided into deep SWI (DSWI) and superficial SWI (SSWI). In recent years, the use of 18F-Fluorodeoxyglucose (FDG)-positron emission tomography (PET)/computed tomography (CT) in diagnosing infections and inflammation has expanded significantly, with a growing number of clinical indications. This study assesses FDG-PET/CT diagnostic role in DSWI detection, the evolution of FDG uptake intensity in patients without DSWI and the potential biomarkers for DSWIs prediction.

**Methods:**

We conducted a single center prospective study of consecutive patients referred for suspected SWI post-median sternotomy. Gold standard diagnosis was established by chart review of clinical follow-up, surgical findings, and cultures. To characterize the time between sternotomy and imaging, participants were subsequently subdivided into recent (< 3 months) or remote surgery (≥ 3 months) groups.

**Results:**

44 FDG-PET/CT scans, 12 (27%) of which had DSWI according to the gold standard, were collected and analyzed. 20 studies were assigned to the recent group, and 24 studies to the remote surgery group. Sensitivity and specificity of FDG-PET/CT for detection of DSWI were 67% and 66%, respectively and an accuracy of 66% was obtained. Positive and negative predictive values were 42% and 84%, respectively. The NPV was higher in the remote surgery group (100%) compared to the recent surgery group (73%). SUV_max_ of the median sternal wound was significantly higher in the DSWI (9.3 ± 2.3) than the non-DSWI group (7.1 ± 3.0) (*p* = 0.025). There was however significant overlap of SUV_max_ between the two groups. CRP, WBC counts, and PCT levels were not significantly different between the DSWI and non-DSWI groups (*p* ≥ 0.34).

**Conclusion:**

FDG-PET/CT has modest sensitivity and specificity for the detection of DSWI post-sternotomy. FDG-PET/CT results must take into account time since surgery; when PET/CT is performed more than 3 months following surgery, a negative scan can exclude DSWI with a high level of certainty.

## Introduction

Sternal wound infections (SWIs) are an uncommon but potentially life-threatening complication of median sternotomy. Delayed diagnosis may result in increased morbidity (Braxton et al. 2000; Lazar et al. 2016; Milano et al. 1995). While diagnosis of SWI relies mostly on clinical and para-clinical findings such as erythema, fever, purulent drainage, sternal instability and elevated inflammatory biomarkers, imaging can help differentiate between superficial SWIs (SSWIs) and deep SWIs (DSWIs), as well as monitor response to therapy. SSWIs are more common and involve only the skin, subcutaneous tissues, and/or pectoralis fascia. Distinct of these are DSWIs, which extend to the sternum, substernal space and/or mediastinum, causing osteitis or mediastinitis. As medical treatment, surgical management and clinical outcomes differ between the two (Lazar et al. 2016), early differentiation between SSWIs and DSWIs is essential.

Computed tomography (CT) is often the first-line imaging technique used for the identification of suspected sternal wound complication. However, recent median sternotomy procedures can lead to normal post-operative changes until at least one year post-surgery, including pericardial effusion and retrosternal fluid collection (Blomjous et al. 2023). These normal inflammatory post-operative changes can decrease the CT ability to detect infection. Magnetic Resonance Imaging (MRI) is a highly effective imaging method for detecting early soft tissue, muscle and bone involvement. MRI is considered to have a higher accuracy than CT for the diagnosis of early stages of suspected osteomyelitis as marrow edema presents as hypointensity on T1-weighted images and increased T2 signal (Hota et al. 2018). MRI, however, is susceptible to artifacts from metallic sternal wires that hamper depiction of surrounding soft tissue and bone in the postoperative period and may be contraindicated in the presence of epicardial leads. In the diagnosis of active osteomyelitis, an accuracy of MRI of 81% was reported against 85% for FDG PET/CT (Demirev et al. 2014).

Fluorodeoxyglucose-positron emission tomography/CT (FDG-PET/CT) is established as a useful imaging modality in numerous conditions such as endocarditis, vascular graft infections, osteomyelitis, and fever of unknown origin (Mahmoodi et al. 2022; Palestro et al. 2021; Treglia et al. 2020; Lucinian et al. 2020; Pelletier-Galarneau et al. 2020; Rijsewijk et al. 2023; Abikhzer et al. 2022; Li et al. 2018 Mar). Recent retrospective studies suggest that FDG-PET/CT is an accurate modality to detect SWI and differentiate between SSWIs and DSWIs (Hariri et al. 2019; Zhang et al. 2018). The purpose of this prospective study is to evaluate the diagnostic accuracy of FDG-PET/CT to detect DSWI, to assess the temporal evolution of FDG uptake intensity in patients without DSWI, and to examine the predictive value of commonly used biomarkers of infection in detecting DSWIs.

## Methods

### Study population

Consecutive patients aged 18 years or older referred to the Nuclear Medicine Department of the Montreal Heart Institute between February 2019 and May 2023 for an FDG-PET/CT study for suspicion of SWI were prospectively enrolled. Only patients with body mass index superior to 45 kg/m^2^ were excluded from the study.

### FDG-PET/CT imaging

All patients underwent whole-body FDG-PET/CT scans performed on a hybrid PET-CT scanner (Siemens Biograph mCT Flow 40 with TrueV, Knoxville, TN, USA). Standard imaging protocol included a myocardial suppression preparation with a 24-h low-carbohydrate high-fat diet, > 12 h fasting, and low-dose heparin (50 UI/kg) 15 min prior to FDG injection. Approximately 370 MBq of ^18^F-FDG was injected intravenously and 60 min after whole-body PET-CT imaging from the base of the skull to proximal femur were acquired in 3D-mode. A low-dose CT scan without contrast was obtained after PET acquisition for attenuation correction and anatomic correlation. PET images were reconstructed using ordered-subset expectation maximization (2 iterations and 21 subsets) algorithm with time of flight and point spread function modeling (ultraHD, Siemens). A 5-mm isotropic Gaussian filter was applied to the reconstructed images.

### FDG-PET/CT interpretation

**Fig. 1:**
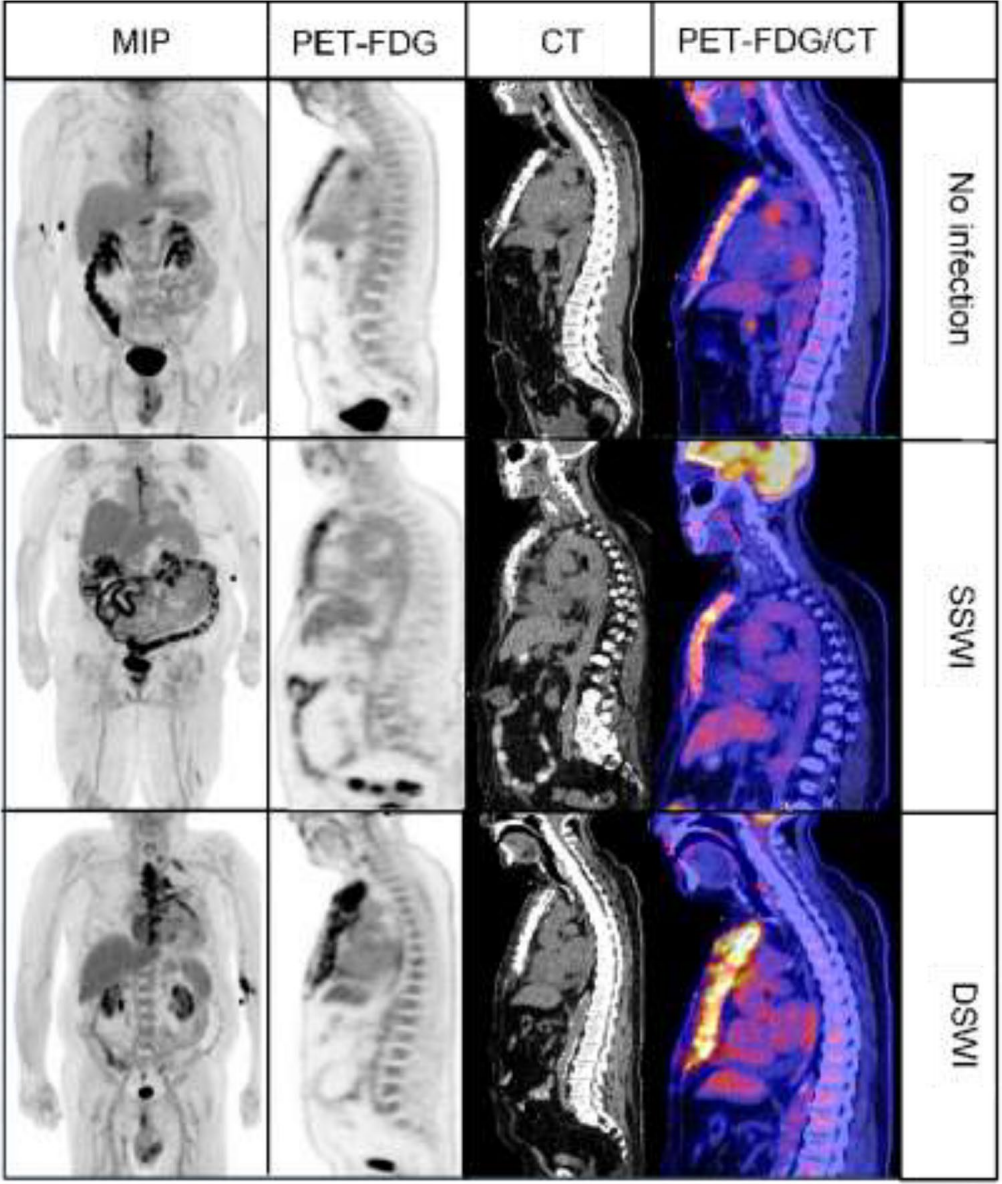
Anterior maximal intensity projection (MIP) images and sagittal PET, CT, and fused PET/CT images of the FDG sternotomy uptake patterns for no infection, superficial sternal wound infections (SSWI), and deep sternal wound infection (DSWI) referring to osteitis and/or mediastinitis

The interpretation of FDG-PET/CT scans was conducted independently by two experienced nuclear medicine physicians. Except for knowledge of time interval between cardiac surgery and PET/CT imaging, interpretation was blinded to all clinical information. Subjective assessment using gestalt interpretation included one of the following five categories: no infection, superficial infection, sternal osteitis, mediastinitis, and absent uptake (Fig. 1). Disagreements were resolved by consensus. In addition to gestalt interpretation, SUV_max_ were measured, and uptake patterns were categorized as previously described (Hariri et al. 2019): soft tissue extension, focal uptake, sternal wire uptake, diffuse high-grade uptake, and diffuse low-grade uptake. Furthermore, FDG uptake of the spleen in comparison to that of the liver was measured to calculate a spleen-to-liver ratio. Finally, specific findings of infection CT scans including presence of osseous resorption, subcutaneous emphysema, collection, mediastinal edema, and widened mediastinum, were reported.

### Gold standard

The final clinical diagnosis based on multidisciplinary consensus with a minimum follow-up of 3 months was used as the gold standard to distinguish DSWI from the non-DSWI group, which included the SSWI and non-infected group. Detection of DSWI was confirmed in the presence of positive culture from an excised wire or in the presence of positive surgical findings, such as purulent material around the sternum or wires in subjects who underwent surgery. DSWI was also considered present in subjects with a purulent drainage from thoracic drains. The non-infected group comprised of those with negative cultures and surgical findings, and those who did not undergo sternal debridement and did not show evidence of sternal infection on clinical follow up.

### Statistical analysis

Categorical variables were presented as frequencies and continuous variables as mean ± SD with the exception of time interval between sternotomy and PET/CT which is presented as median (interquartile range). Categorical variables were compared using chi-square tests when there were more than two categories or Fisher’s exact test when there were two categories. Continuous variables were compared with Student’s two-sample t test. A p value of < 0.05 was considered significant. Inter-observer agreement was analyzed using the Cohen's Kappa coefficient. AUC statistics for ROC curves were measured with the Mann–Whitney U statistic. Specificity, sensitivity, and accuracy of our interpretation criteria with FDG-PET/CT were compared to that of SUV_max_ using a McNemar's test. The optimal cut-off value for SUV_max_ was calculated using the Youden's index. Participants were divided into recent (< 3 months between sternotomy and imaging) or remote surgery (≥ 3 months between sternotomy and imaging) for subgroup analyses. All analyses were performed using SAS Studio (SAS Studio 3.8 on SAS 9.4 for Windows, North Carolina USA, www.welcome.oda.sas.com) and GraphPad Prism (GraphPad Prism version 10.0.2 for Windows, GraphPad Software, La Jolla California USA, www.graphpad.com).

## Results

### Population characteristics

A total of 44 FDG-PET/CT studies in 40 patients acquired between February 2019 and May 2023 were included. Of the 44 cases, 12 (27.3%) had a final diagnosis of DSWI and 32 cases (72.7%) had no DSWI according to the gold standard. Of the 44 studies included, 20 (45.5%) were performed within 3 months after surgery and were assigned to the recent surgery subgroup while 24 (55.5%) were performed more than 3 months after surgery and were assigned to the remote surgery subgroup. Of the 12 patients with DSWI, 7 (58.3%) were in the recent surgery subgroup, and 5 (41.7%) were in the remote surgery subgroup.

Participants’ age ranged from 21 to 83 years with average age of 63 ± 13 years. The study population was predominantly male (30/40, 75.0%). The median time between PET/CT and surgery was 3.9 months (IQR 1.4–8.1). The median time interval between imaging and surgery for those with DSWI was 2.2 months (0.8–6.3, range 17 days to 2.3 years) and 4.4 months (1.8–8.2, range 13 days to 2.4 years) for those without DSWI (*p* = 0.32). Participants with DSWI had on average lower BMI compared to the non-DSWI group (26.9 ± 5.1 vs 31.2 ± 4.5, kg/m^2^, *p* = 0.010) and were older (70 ± 9 vs 61 ± 13 years, *p* = 0.042). C-reactive protein (CRP), white blood cell (WBC) counts, and procalcitonin (PCT) levels were not significantly different between those with and without DSWI (*p* ≥ 0.34). Patient demographics and baseline characteristics were similar between those with and without DSWI (Table 1).

**Table 1:** Baseline characteristics of all included subjects

	All cases (n = 44)	DSWI (n = 12)	No DSWI (n = 32)	*p* value
Age ± SD (years)	63 ± 13	70 ± 9	61 ± 13	0.042
Female (n, %)	12 (27.3)	3 (25.0)	9 (28.1)	0.84
BMI (kg/m^2^)	30.0 ± 5.0	26.9 ± 5.1	31.2 ± 4.5	0.010
Time since sternotomy (months, IQR)	3.9 (1.4–8.1)	2.2 (0.8–6.3)	4.4 (1.8–8.2)	0.32
Surgery (n, %)				0.26
Aortic surgery	2 (4.6)	2 (16.7)	0	
CABG	26 (59.1)	9 (75.0)	17 (53.1)	
Heart transplant	4 (9.1)	0	4 (12.5)	
LV aneurysm repair	1 (2.3)	0	1 (3.1)	
Congenital	1 (2.3)	0	1 (3.1)	
Valve replacement	1 (2.3)	0	1 (3.1)	
CABG and valve replacement	2 (4.6)	0	2 (6.3)	
Ross procedure and aortic surgery	1 (2.3)	0	1 (3.1)	
Valve replacement and aortic surgery	6 (13.6)	1 (8.3)	5 (15.6)	
Mammary artery (n, %)				0.45
0	16 (36.4)	3 (25.0)	13 (40.6)	
1	19 (43.2)	7 (58.3)	12 (37.5)	
2	9 (20.5)	2 (16.7)	7 (21.9)	
CRP ± SD (mg/L)	62.5 ± 69.1	80.3 ± 67.6	55.3 ± 69.7	0.34
WBC ± SD (× 10^9^/L)	8.1 ± 3.1	7.8 ± 4.7	8.2 ± 2.2	0.71
Procalcitonin ± SD (ug/L)	0.18 ± 0.097	0.18 ± 0.11	0.18 ± 0.097	0.92

*BMI* body mass index, *CABG* coronary artery bypass, *CRP* C-reactive protein, *LV* left ventricle, *WBC* white blood cells

### FDG-PET/CT imaging findings

FDG-PET/CT findings for all participants are summarized in Tables 2 and 3. Focal uptake, uptake extending in surrounding soft tissue, subcutaneous emphysema, mediastinal edema, osseous resorption, and soft tissue collection were all associated with DSWI, but the association did not reach statistical significance. No combination of FDG-PET/CT findings were found to significantly improve accuracy. Diffuse low grade/absent uptake and sternal wire uptake were associated with absence of DSWI.

**Table 2:** Sternotomy site imaging findings on PET/CT

Imaging features	Total (*n* = 44)	DSWI (*n* = 12)	No-DSWI (*n* = 32)	*p* value
*FDG patterns of uptake*				
SUV max sternum ± SD	7.7 ± 2.9	9.3 ± 2.3	7.1 ± 3.0	0.025
SUV max mediastinum ± SD	4.9 ± 4.0	7.6 ± 5.3	3.8 ± 2.9	0.036
Soft tissue extension	23 (52.3%)	8 (66.7%)	15 (46.9%)	0.32
Focal uptake	21 (47.7%)	8 (66.7%)	13 (40.6%)	0.18
Sternal wire uptake	4 (27.3%)	0 (0.0%)	4 (12.5%)	0.56
Diffuse high-grade uptake	26 (59.1%)	7 (58.3%)	19 (59.4%)	1.00
Diffuse low-grade uptake	7 (15.9%)	1 (8.3%)	6 (18.8%)	0.65
Absent uptake	2 (4.6%)	0 (0.0%)	2 (6.3%)	1.00
*CT findings*				
Osseous resorption	4 (9.1%)	2 (16.7%)	2 (6.3%)	0.30
Subcutaneous emphysema	7 (15.9%)	4 (33.3%)	3 (9.4%)	0.075
Collection	18 (40.9%)	7 (58.3%)	11 (34.4%)	0.18
Mediastinal edema	16 (36.4%)	7 (58.3%)	9 (28.1%)	0.086

**Table 3:** Diagnostic performances of individual PET and CT imaging findings

Imaging features	OR (95% CI)	Sensitivity (95% CI)	Specificity (95% CI)
*PET findings*			
Focal uptake	2.9 (0.7–11.8)	66.7 (40.0–93.3)	59.4 (42.4–76.4)
Hepatosplenic inversion	2.3 (0.4–12.5)	25.0 (0.5–49.5)	87.5 (76.0–99.0)
Soft tissue extension	2.3 (0.6–9.1)	66.7 (40.0–93.3)	53.1 (35.8–70.4)
Sternal wire uptake	-	12.5 (1.0–24.0)	100.0 (100.0–100.0)
Diffuse high-grade uptake	1.0 (0.2–3.7)	58.3 (30.4–86.2)	40.6 (23.6–57.6)
Diffuse low-grade uptake	0.4 (0.04–3.7)	8.3 (0.0–24.0)	81.3 (67.7–94.8)
Absent uptake	-	6.3 (0.0–14.6)	100.0 (100.0–100.0)
*CT findings*			
Subcutaneous emphysema	4.8 (0.9–26.2)	33.3 (6.7–60.0)	90.6 (80.5–100.0)
Mediastinal edema	3.6 (0.9- 14.3)	58.3 (30.4–86-2)	71.9 (56.3–87.5)
Osseous resorption	3.0 (0.4–24.1)	16.7 (0.0–37.8)	93.8 (85.4–100.0)
Collection	2.7 (0.69- 10.4)	58.3 (30.4–86.2)	65.6 (49.2–82.1)

The proportion of patients with inversion of spleen-to-liver ratio (SLR) was not significantly different between the DSWI and the non-DSWI groups (3/12, 25% vs 4/32, 12.5%,* p* = 0.37). The SLR was also not significantly different in the recent surgery group and the remote surgery group between the DSWI and the non-DSWI groups (2/7, 28.6% vs 4/13, 30.8%, *p* = 1.00) and (1/4, 25.0% vs 0/19, 0.0%, *p* = 0.21) respectively.

### Post-operative sternal FDG uptake and SUVmax

**Fig. 2:**
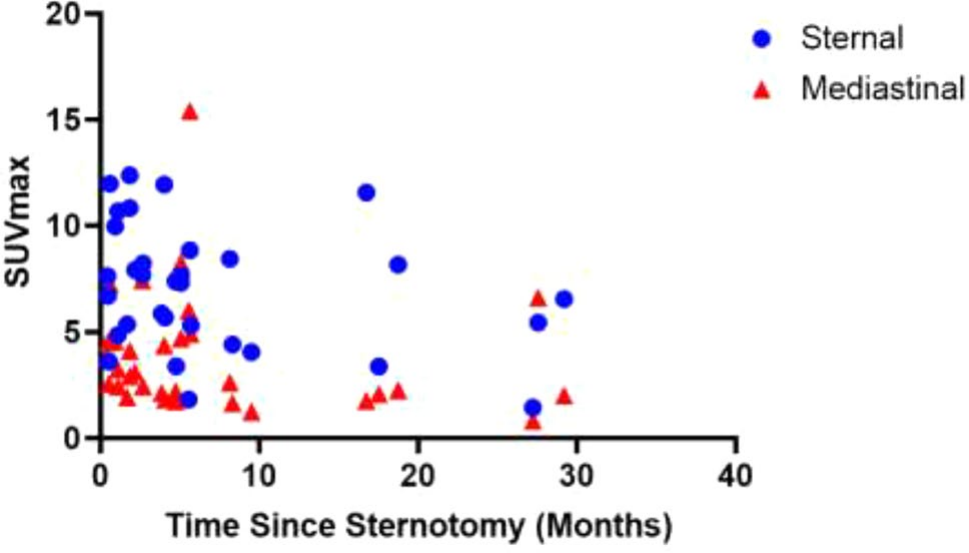
Mediastinal and sternal SUV_max_ of non-DSWI patients as a function of time since sternotomy

Evolution of sternal and mediastinal uptake following surgery is shown in Fig. 2. For the non-DSWI group, in the early postoperative period (< 3 months), the mean SUV_max_ at the sternotomy site was 8.3 ± 2.8 (range, 3.6–12.4) vs 6.2 ± 2.9 (range, 1.4–11.9) in the late postoperative period (≥ 3 months). The mean SUV_max_ of all non-DSWI in the mediastinum was found as 3.9 ± 1.7 (range, 1.9–7.4) in the recent surgery subgroup vs 3.8 ± 3.5 in the remote surgery subgroup (range, 0.83–15.4).

**Fig. 3:**
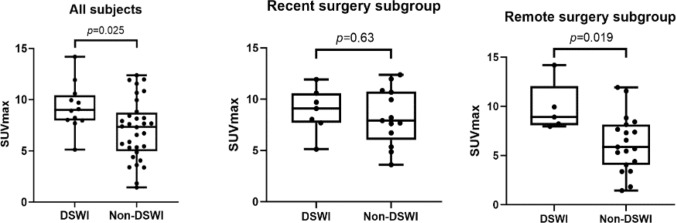
Distribution of sternal SUV_max_ in the DSWI and non-DSWI participants, in all subjects, and in recent (< 3 months) and remote (≥ 3 months) surgery subgroups

SUV_max_ of median sternal wound was significantly higher in the DSWI group compared to the non-DSWI participants (9.3 ± 2.3 vs 7.1 ± 3.0, *p* = 0.025). In the recent surgery group, the sternal uptake was non-significantly higher in the DSWI group compared to non-DSWI participants (8.9 ± 2.2 vs 8.3 ± 2.8, *p* = 0.63). Conversely, the association is significant in the remote surgery group with the SUV_max_ higher in the DSWI group compared to non-DSWI group (9.9 ± 2.5 vs 6.2 ± 2.9, *p* = 0.019). SUV_max_ of the mediastinal tissues is similarly higher in the DSWI subjects (7.6 ± 5.3) compared to non-DSWI subjects (3.8 ± 2.9) and the difference was statistically significant in the overall (*p* = 0.036) and recent surgery group (6.1 ± 2.9 vs 3.9 ± 1.7, *p* = 0.046), but not in the remote surgery group (9.7 ± 7.4 vs 3.8 ± 3.5, *p* = 0.1531). The ROC curve demonstrated that a SUV_max_ cutoff value of 7.66 yielded a sensitivity of 91.7% (95%CI: 64.6–99.6%) and a specificity of 59.4% (95%CI: 42.3–74.5%). However, there was a significant overlap of SUV_max_ range across the DSWI and non-DSWI groups, yielding no clinically useful threshold value to determine a deeper infection (Fig. 3).

### Diagnostic accuracy

Gestalt interpretation yielded an overall modest accuracy of 66% (95% CI 52–80%) with a sensitivity and specificity of 67% (95% CI 40–93%) and 66% (95% CI 49–82%), respectively, for the diagnosis of DSWI when compared to the gold standard (Table 4). In the recent surgery group, the diagnostic accuracy of PET/CT was 70% (95% CI 50–90%) with a sensitivity and specificity of 43% (95% CI 6–80%) and 85% (95% CI 65–100%), respectively. In the remote postoperative period subgroup, the diagnostic accuracy was 63% (95% CI 43–82%) with a sensitivity and specificity of 100% (95% CI 100–100%) and 53% (95% CI 30–75%), respectively. The specificity was significantly higher in the remote surgery group compared to the recent surgery group (*p* = 0.021) and sensitivity was not significantly different (*p* = 0.74). The positive predictive value (PPV) and negative predictive value (NPV) were 0.42 (95%CI: 0.20–0.64) and 0.84 (95%CI: 0.70–0.98), respectively. The NPV was higher in the remote surgery group (1.00) compared to the recent surgery group (0.73). Findings demonstrated moderate agreement between the nuclear medicine physicians (*κ* = 0.55, 95% CI 0.31–0.79). Interobserver agreement was poor in the early post-operative period (κ = 0.18, 95% CI − 0.25 to 0.60) and very good in the late post-operative period (κ = 0.83, 95% CI 0.61–1.00.

**Table 4:** Two-by-two diagnostic table for the diagnosis of deep sternal wound infections using the gestalt FDG-PET/CT interpretation

	Gold standard	
	DSWI	Non-DSWI
*FDG-PET/CT*		
DSWI	8	11
Non-DSWI	4	21

ROC analyses of gestalt interpretation of FDG-PET/CT scans yielded an AUC of 0.66 ± 0.083 (95% CI 0.50–0.82, *p* = 0.051). In the recent surgery setting, ROC curve analysis of consensus interpretation yielded an AUC of 0.64 ± 0.11 (95% CI 0.41–0.86, *p* = 0.23) compared to an AUC of 0.76 ± 0.059 (95% CI 0.65–0.88, *p* = < 0.00001) in the remote surgery setting. In our analysis of predictors, age, time since sternotomy, CRP, WBC and PCT levels were not significantly associated with a false positive or a false negative FDG-PET/CT for DSWI diagnosis.

## Discussion

The role of FDG-PET/CT in the evaluation and diagnosis of cardiovascular infections such as endocarditis and device infections has expanded over the last years (Martineau et al. 2021), but data on FDG-PET/CT accuracy remains limited in several indications, including SWI. This study is the largest prospective studies evaluating the diagnostic performance of PET in SWI. We found that FDG-PET/CT showed a modest diagnostic accuracy of 66% (95% CI 52–80%), sensitivity of 67% (95% CI 40–93%), and specificity of 66% (95% CI 49–82%) for detecting DSWI. FDG-PET/CT has an overall very good NPV of 0.84 and excellent NPV in patients imaged ≥ 3 months after surgery (1.00). The sensitivity, specificity and accuracy are suboptimal, but still provide instrumental information in challenging patients in a clinical setting.

There is limited literature addressing the utility of FDG-PET/CT in patients with suspected DSWI after cardiac surgery. The sensitivity and specificity of CT has been reported to be 88% and 91%, respectively, for mediastinitis, and 93% and 85–96%, respectively, for osteomyelitis of the posterior and anterior sternal plate in the weeks following surgery (Gur et al. 1998). However, in clinical practice, CT findings are often nonspecific in the immediate post surgery period and follow up CT is often required to confirm the diagnosis.

In a retrospective study of 73 patients, sensitivity and specificity of PET-CT were 98% and 78% for sternal osteomyelitis, 100% and 95% for mediastinitis, and 82% and 100% for costochondritis (Zhang et al. 2018). Our group previously showed that FDG-PET is accurate for the detection of SWI and proposed interpretation criteria which rely on tracer uptake distribution rather than uptake intensity (Hariri et al. 2019). We reported an excellent sensitivity and specificity of 91% and 97% respectively with an area under the curve (AUC) of the receiver-operator characteristic (ROC) analysis of 0.96 while use of SUV_max_ yielded an AUC of 0.82 with a sensitivity and specificity of 100% and 69% respectively at a cutoff of 6.5. The results supported that interpretation based on imaging patterns was associated with improved specificity, especially in the recent surgery subgroup (≤ 6 months between sternotomy and imaging).

The results of this study are somewhat discordant with a lower accuracy compared to our previous study. Besides the fact that both studies are relatively small and thus reported sensitivity and specificity values have broad confidence intervals, other factors could account for those differences. Notably, the median time since sternotomy was 42.4 ± 68.2 months for the non-infected group while the overall time for all subjects was 32.6 ± 60.4 months versus 4.4 (1.8–8.2) and 3.9 (1.4–8.1) in this study. Shorter time interval between surgery and PET imaging translates to more extensive postoperative inflammatory response, which may contribute to an increase rate of false positives and a corresponding decrease in specificity.

In our previous work, characteristics of subjects with confirmed post-thoracotomy infection included CT findings of subcutaneous emphysema, widened mediastinum, soft tissue collection, and osseous resorption while PET findings predictor of SWI included sternal wire uptake, soft tissue uptake extending into the sternum and focal uptake (Hariri et al. 2019). However, in this study, no CT or FDG-PET features were found to be statistically significant predictors of DSWI. A relatively lower sensitivity was thus observed in the diagnosis of DSWI because of the absence of typical FDG-PET/CT findings of mediastinitis, osteitis, or abscess in our group and/or a higher rate of these features in the non-DSWI group. Again, this could be because these imaging findings are not specific in the immediate post-surgery period. This further emphasize the importance to take into account time since surgery; when PET/CT is performed more than 3 months following surgery, a negative scan can exclude DSWI with a high level of certainty.

### ^18^F-FDG uptake intensity

The ^18^F-FDG uptake intensity was significantly higher in the DSWI group at the sternotomy site and mediastinum. The difference in sternal uptake was not statistically significant in the recent surgery group, but it was statistically significant in the remote surgery group. This indicates that normal postoperative inflammation can limit the specificity in the early post-operative period. This is consistent with a previous study demonstrating increased ^18^F-FDG uptake **up to** 1-year post-surgery in 45% of healthy patients without DSWI (Blomjous et al. 2023).

In our study, defining a diagnostic SUV_max_ cut-off value was not possible due to significant overlap between participants with and without DSWI. As previously mentioned, diffuse high-grade uptake and retrosternal collection were the most common PET/CT findings in the non-DSWI subjects which may reflect normal healing process and postoperative changes.

Interestingly, besides FDG uptake intensity, only age and BMI were associated with increased risk of DSWI. Serum markers of infection and inflammation including CRP, WBC, and PCT were not different between those with and without DSWI. Of the 20 patients without SWI, 6 had a differential diagnosis of infectious or inflammatory origin (pericarditis, dental abscess or anorectal abscess). This may explain the non-significantly higher levels of CRP and the similar levels of WBC and PCT in the DSWI group compared to the group with no suspected sternal infection and SSWI.

Increased splenic FDG uptake is associated with a systemic inflammatory response and can be indicative of cardiovascular infection (Pijl et al. 2021). However, in this study, increased splenic uptake was not a marker of DSWI. This is likely related to the non-specificity of increased splenic uptake, which can be observed in various infectious or inflammatory conditions. In addition, in the context of recent surgery, CRP levels and increased splenic uptake is frequently observed due to post-surgery inflammation.

## Limitations

The specificity of FDG-PET in this study may be underestimated due to limitations in the gold standard. For instance, negative perioperative cultures, coupled with the subsequent use of a vacuum-assisted closure (VAC) device, may result in misclassifying a patient as non-DSWI when an infection is actually present. The study has a small sample size, resulting in broad confidence intervals and limiting our ability to reach definite conclusions about the significance of the different imaging features. Nonetheless, this is the largest prospective study on FDG-PET and SWI. At our center, patients with definite diagnosis are unlikely to undergo FDG-PET imaging in order to limit treatment delays. This may lead to a referral bias towards more complicated patients, and a higher or lower disease prevalence and a selection bias could be introduced. Nonetheless, the study population is likely more representative of actual clinical practice where patients have no clear diagnosis or discordant investigations, thereby providing real-world data closer to the true diagnostic performance in the clinical setting.

## Conclusion

FDG-PET/CT has an excellent NPV of 0.84 and a modest diagnostic accuracy, sensitivity and specificity to detect DSWI. Sensitivity and NPV is excellent when imaged 3 months or more after median sternotomy. Although uptake intensity was greater in those with DSWI, the significant overlap between DSWI and non-DSWI patients prevents the use of a definite diagnostic threshold and interpretation should take into consideration uptake distribution. Stand-alone diagnostic biomarkers such as CRP, WBC and PCT are not useful for detecting and differentiating between DSWI and non-infected or SSWI patients. Further research is needed to establish predictors of false positive and false negative FDG-PET/CT results for the accurate diagnosis of SWI and to define the prognostic value of FDG-PET in this population.

## Data Availability

The datasets generated during and/or analysed during the current study are available from the corresponding author on reasonable request.
